# Microbending Experiments on Pure Magnesium with Nonbasal Slip Orientation

**DOI:** 10.3390/ma11081434

**Published:** 2018-08-14

**Authors:** Jan Maňák, David Vokoun

**Affiliations:** Institute of Physics ASCR, Na Slovance 2, CZ-182 21 Praha 8, Czech Republic; vokoun@fzu.cz

**Keywords:** magnesium, microcantilevers, microbending, focused ion beam (FIB)

## Abstract

In the present study, in situ microbending experiments on magnesium single crystalline microcantilevers are presented. Microcantilevers with pentagonal cross-section were fabricated by focus ion beam. Two basic crystallographic orientations of the microcantilevers were investigated: {0001} and {10-10}, i.e., the *c*-axis perpendicular to and parallel with the cantilever top surface, respectively. After bending, the longitudinal sections of the microcantilevers were analyzed using electron backscatter diffraction to investigate the crystal lattice rotations and accumulated deformations. The stress levels in the loaded cantilevers are strongly dependent on the crystal orientation. Extension twins were found in the {10-10} cantilevers.

## 1. Introduction

Magnesium is a lightweight material. Due to its low density and high strength-to-weight ratio and as well as its potential for a wide range of applications [1,2], magnesium has received a great deal of attention. One of the serious obstacles in using magnesium is its anisotropic behavior. In order to characterize the anisotropic mechanical properties, or deformation mechanisms, mechanical properties of Mg single crystals have been studied since the beginning of the search for lightweight materials [3,4,5,6,7]. During macroscopic sample preparation, some unattended and undesirable deformations may occur, which then complicate these studies of the deformation mechanisms. Recent advances in in situ mechanical tests on micrometric samples, micromachined using SEM/FIB (scanning electron microscope/focused ion beam), have yielded methods offering strong advantages over the traditional mechanical testing methods [8]. Furthermore, with emerging small-scale engineering applications, such as microelectromechanical systems and various microcomponents, the mechanical properties of magnesium at micro-scale are of interest. Since mechanical properties at micro- and macro- scale may differ due to various size-scale phenomena [9,10,11], further studies are necessary to determine the possibility of using miniature magnesium components for biomedical and aerospace applications [12,13].

Several research groups have analyzed mechanical properties of magnesium at micro-scale, mostly working with nano- and micro-pillars [8,14,15,16]. To our knowledge, microbending experiments on pure single crystal magnesium have not been reported before. Most of microbending experiments were performed with fcc (face-centered cubic) or bcc (body-centered cubic) metals and alloys [17,18,19]. Metals with hcp (hexagonal closed-packed) crystalline structures have a reduced number of available slip systems compared to fcc and bcc metals, which make plastic deformation more difficult [20,21]. Generally, there are multiple purposes for microbending experiments and simulations with metallic materials: (i) The study of the size-effects; (ii) gaining deeper insight into microplasticity; (iii) validation of various micro-mechanical models [10,22,23].

In the present study the effect of the crystallographic orientation of Mg on the stress-deflection curves obtained from microbending was considered without regard for the size-effects.

## 2. Materials and Methods

**Figure 1:**
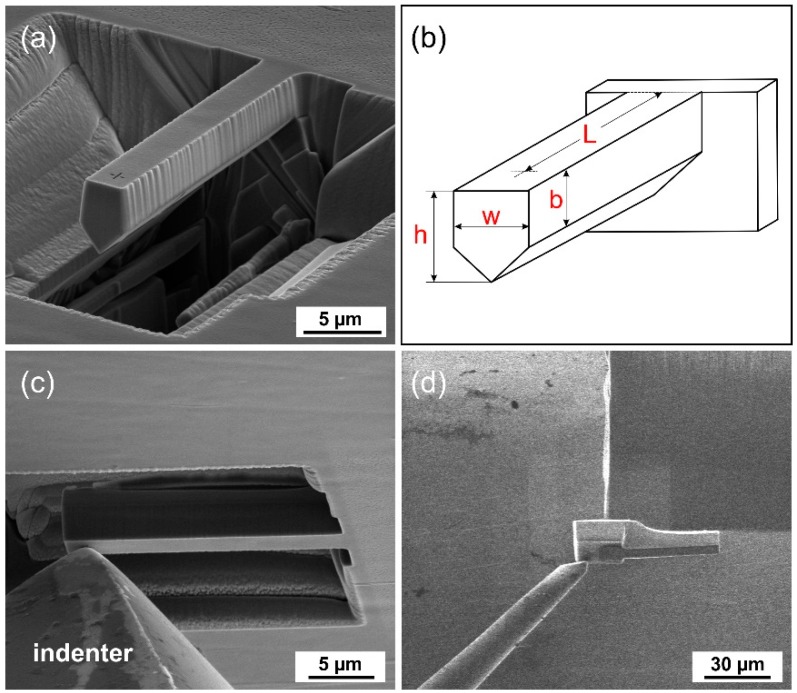
Microcantilever with pentagonal cross-section (**a**), schematic definition of geometric parameters (**b**) in situ microbending (**c**) and a cut-out of micro-cantilever (**d**).

Pure magnesium single crystal was produced using a Bridgman technique and oriented by XRD (X-ray Diffraction, Seifert ISO-Debyeflex 3003, XRD Eigenmann GmbH, Hormersdorf, Germany) measurements and then cut into samples with desired crystallographic orientation with the experimental error less than 1°. The sample went through a standard metallographic preparation followed by electro-polishing. The microcantilevers were micro-fabricated and observed using an FEI Quanta 3D Dual-Beam SEM/FIB (scanning electron microscope/focused ion beam) system (FEI, Hillsboro, USA) with a Ga^+^ ion source operated at 30 keV with various currents using automated milling script. In order to suppress the basal slip, the orientations of Mg in the microcantilevers were selected so that the basal plane was parallel or perpendicular to loading [15]. Hence the chosen orientation arrangements were favorable for the activation of nonbasal slip systems. The crystallographic orientation arrangements, denoted A, B, C, or D, of the microcantilevers, also denoted as A, B, C, or D, correspondingly, are summarized in Table 1. Maximum tensile stresses, *s*_max_, due to bending (see Table 1) were computed from four data sets obtained from each orientation of the microcantilevers according to formula 2 from a past study [24] (*s* = *PLy*/*I*, where *P* is the applied bending force, *L* is the distance between the fixed end and the point where the force is applied, *y* is the vertical distance between the upper surface and the neutral plane, and *I* is the moment of inertia of the beam cross-section). Then *s*_max_ = *P*_max_*Ly*/*I*, where *P*_max_ is the maximum measured force. Moment *I* depends on the geometry of the cross-sectional area of the individual microcantilevers. The in situ microbending tests were performed using a Hysitron PI 85 SEM PicoIndenter (Hysitron, Minneapolis, USA) with a cono-spherical diamond tip of 1 μm diameter. The crystallographic orientations of micro-cantilever longitudinal sections were acquired using the FEI Quanta 3D equipped with an EDAX Hikari EBSD (electron backscatter diffraction) detector (EDAX, New Jersey, USA) and analyzed with a help of EDAX OIM software (version 8). Finite element analysis (FEA) was performed using Comsol Multiphysics Software (version 5.3a) [25]. Figure 1a shows one of the micro-fabricated cantilevers. Dimensions of samples A, B, C, and D defined in Figure 1b and summarized in Table 2 are not far from the prescribed dimensions (length × height × depth = 22 μm × 4.5 μm × 3 μm). As for the milling procedure, an automated script with 8-steps preparation process was made. The milling currents were ranging from 15 nA (roughing) to 3 nA (finishing). Figure 1c shows in situ microbending. Microcantilevers A, B, C, and D were bent with constant loading rate 50 nm/s under displacement control mode. The simultaneous SEM observation helped maintain a precise placement of the indenter’s tip. After the loading tests, it was necessary to make several cuts in order to prepare samples for the EBSD measurements. Before that, the samples were coated with a protective *W* layer to reduce ion damage of the microcantilevers during cutting. Figure 1d shows a cut-out (including the microcantilever) from the bulk sample. The cut-out was transferred and fixed to the edge of the sample with a micromanipulator. Longitudinal sections of the microcantilever were cut away. The cuts were made along the micro-cantilever axis using milling current as low as 1 nA to minimize any damage. As for EBSD data acquisition, each sample was tilted to obtain the desired angle of tilt required for EBSD measurements. The EBSD maps were measured with a 200 nm step size. 

## 3. Results

**Figure 2:**
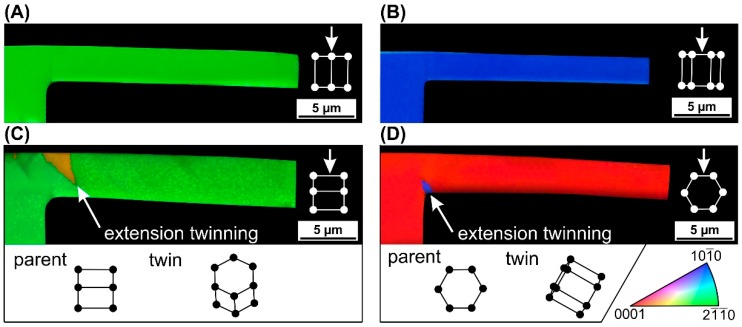
The electron backscatter diffraction (EBSD) analysis of the longitudinal sections of the cantilever samples. The labels (**A**–**D**) correspond to the sample crystallographic orientations (A-D). All the crystal-structure schematics are shown from lateral view of a selected microcantilever. The arrows represent the direction of the acting force.

Figure 2 shows the EBSD analysis of the longitudinal sections of the cantilever samples. The results are presented by IPF (inverse pole figure) maps combined with IQ (image quality) maps. The basal plane orientations A and B show no slip or twinning mechanism in the volume. The pyramidal slip should be the favorable deformation mechanism but higher stresses are needed for its activation. The prismatic plane orientation C reveals extension twin {10-12} in the tension zone near to the fixed end. The prismatic plane orientation D exposes extension twin {10-12} in the compression zone near to the fixed end. Extension twinning results in the reorientation of the original lattice of the microcantilever by an angle of ~86°.

**Figure 3:**
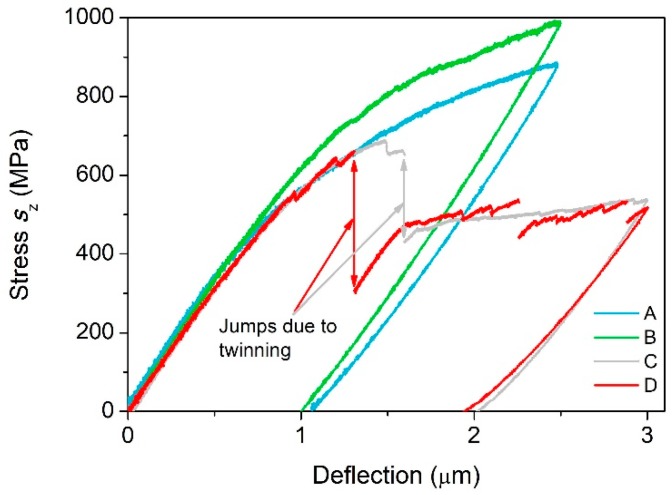
The diagram of stress *s*_z_ versus deflection for representative samples A, B, C, and D.

Figure 3 shows the maximum tensile stress due to bending, *s_z_*, versus deflection for samples A, B, C, and D (*z* is the longitudinal axis of the microcantilever). Stress *s_z_* was obtained from formula 2 from a past paper [24] (*s* = *PLy*/*I*). The curves associated with samples A and B show high stresses and no stress drop. On the contrary, the curves associated with samples C and D display lower stresses and a significant stress drop suggesting ongoing twinning deformation mechanism.

## 4. Discussion

Generally, the stress distribution in the bent cantilevers is of three kinds: (i) Tension stress prevails (in the tension zone), (ii) compressive stress prevails (in the compression zone of the cantilevers), and (iii) no stress component prevails (e.g., the corner area of the fixed end of the cantilever). If the other stress components in the tension and compression zones are neglected, Schmidt factors can be expressed easily for each slip/twinning system. Table 3 summarizes the largest Schmidt factors *m*_1_ calculated according to a past paper [26] and the corresponding slip/twinning systems for either zone of each cantilever sample.

In agreement with Table 3, extension twins were observed in the tension zone of sample C (see Figure 2) and contraction twins were observed in no sample. However, in disagreement with Table 3, no extension twin was observed in the compression zone of sample A, although extension twinning usually has the lowest critical resolved shear stress among the possible slip/twinning systems [27].

Generally, {10-12} <10-1-1> extension twining, the most commonly occurring twinning mode in Mg [28], is the most favorable when a tensile stress is acting along the c-axis of Mg crystal [29]. On the other hand, {10-11} <10-12> contraction twinning occurs only in some cases such as high strain rate when a compressive stress is acting along the c-axis of Mg crystal [8,30]. It is worth mentioning that deformation twinning is inhibited in polycrystalline Mg alloy samples with grain refinement less than 3 μm as fine grains do not satisfy critical twinning stress before slip occurs [31]. However, such grain size does not apply to samples A, B, C, and D. The influence of grain size and other factors (including Schmidt factors) on deformation twinning is studied using statistical analyses in a past paper [32]. In the case of orientation C, the *c*-axis of Mg crystal is parallel with the cantilever axis. Therefore, the extension twins form in the tension zone of the cantilever (Figure 2C). In the case of orientation D, both tensile and compressive stresses are applied perpendicularly to the c-axis of Mg crystal. According to a past paper [29], forming extension twins is suppressed in the tension zone in such a case. As for forming extension twins in the corner part of the fixed end of cantilever D, their formation cannot be easily predicted because of no stress component prevails in this area, i.e., the loading is multi-axial.

Figure 2 also shows that all the longitudinal sections are bent, perhaps due to inelastic deformation (it is worth of noting that the shapes shown do not truly express inelastic deformation of the samples because of several reasons, such as the FIB cut was not precisely parallel with the cantilever axis.) The probable presence of inelastic deformation may indicate local crystal misorientations as a result of dislocation motion. Therefore, Kernel Average Misorientation (KAM) analysis was additionally carried out (the KAM images are not shown in this study). The analysis reveals that the largest misorientations (up to 2°) appear near to the fixed end, forming a narrow band (samples A and B) and at boundaries of the extension twin areas (samples C and D). The observed misorientations are likely a result of dislocation motion in places of stress concentration and tightly around the extension twin areas.

Generally, bending a microcantilever results in a complex multi-axial loading. Thus, the stress distribution in the cantilever can be determined only by carrying out the FEA. Some models for plastic deformation of hexagonal metals have been developed [33,34], however these models, mostly implemented in the finite-element software Abaqus, require input parameters not available to us currently. Therefore, in this study only elastic loading is analyzed. Figure 3 indicates that deflections equal or less than 0.4 μm certainly induce an elastic response of the cantilever (corresponding to maximum stress about 276 MPa in Figure 3). Therefore, in our FEA model, the prescribed deflection was just 0.4 μm. Furthermore, the following elastic constants *C*_11_ = 58 GPa, *C*_12_ = 25 GPa, *C*_13_ = 20.8 GPa, *C*_33_ = 61.2 GPa, and *C*_55_ = 16.6 GPa taken from a previous paper [35] were input into the FEA. The following value of mass density of Mg, ρ_Mg_ = 1738 kg/m^3^ was used in the FEA.

**Figure 4:**
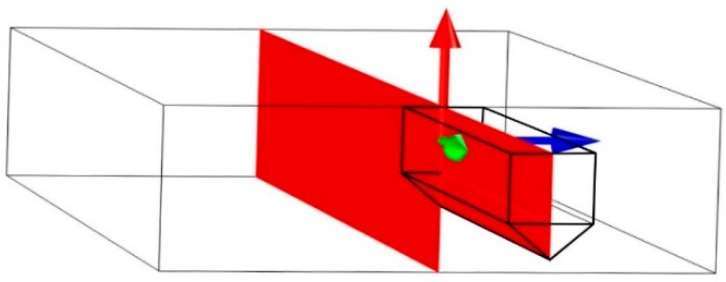
The vertical mirror plane (red color) of the microcantilever.

**Figure 5:**
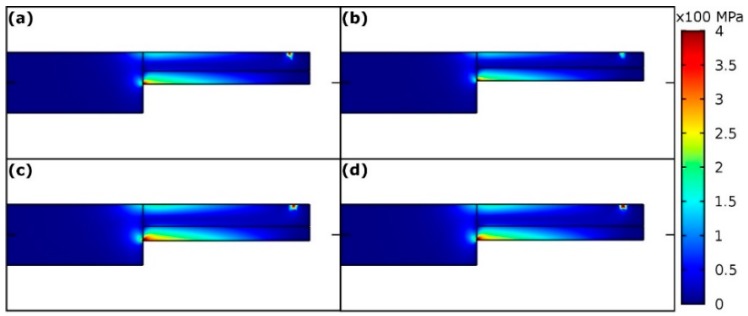
Von Mises stress distribution (in MPa) in the vertical mirror plane of microcantilevers A–D (**a**–**d**), respectively. Cantilever deflection equals 0.4 μm.

Figure 4 shows the vertical mirror plane (red color) of the microcantilever. This plane was chosen for calculation of von Mises stress distribution in the individual microcantilevers (Figure 5) for deflection 0.4 μm. The stress distributions for microcantilevers A, B, C, and D do not differ qualitatively. The largest von Mises stress is always found at the bottom of the fixed end of the microcantilevers. The quantitative differences in the stress distribution among samples A–D are due to: (i) differences in the geometric parameters and (ii) differences in the crystal orientations. In order to see quantitative differences due to various crystal orientations among microcantilevers A, B, C, and D, elastic strain energy density in the fixed bottom corner was evaluated for the fixed cantilever dimensions (*L* = 20 μm, *w* = *b* = 3 μm and *h* = 4.5 μm, which corresponds to the prescribed dimensions) and shown in Table 4. Beside the elastic strain energy density, total elastic strain energy and the calculated {measured} forces corresponding to deflection 0.4 μm are summarized in Table 4. The total elastic strain energy was calculated also for the fixed cantilever dimensions (*L* = 20 μm, *w* = *b* = 3 μm and *h* = 4.5 μm) whereas forces were calculated for the real cantilever dimensions. 

Table 4 indicates that various crystallographic orientation arrangements A, B, C, and D make difference in stress and strain distributions in respective elastically loaded microcantilevers A, B, C, and D. The calculated {measured} forces corresponding to deflection 0.4 μm for cantilevers A, B, C, and D were 62, 42, 104, and 90 μN, {72, 57, 102, and 94 μN}, respectively. A partial reason for the deviation between the calculated and the corresponding measured force values might be the fact that some edges of the microcantilevers were rounded. There is almost a perfect agreement between the calculated and the corresponding measured forces values in the case of samples C and D, whereas a significant disagreement in the case of samples A and B exists. The cross-section areas of samples A and B are smaller than those of samples C and D. Therefore, mechanical properties of samples A and B might be influenced by Ga ion implantation during FIB more than in the case of samples C and D.

## 5. Conclusions

Pure magnesium single crystal was oriented into four various crystallographic orientations in the fabricated microcantilevers either with c-axis perpendicular to or parallel with the cantilever top surface. The stress-deflection curves were obtained from in situ microbending experiments. The basal plane orientations (A, B) showed high bending stresses and no change of crystallographic orientation in the volume. The prismatic plane orientations (C, D) revealed extension twinning. The formation of extension twins in the tension zone of sample C and the absence of contraction twins in all the samples was in agreement with the performed Schmidt factor evaluation. The presented finite element analysis carried out for elastic loading showed (i) that the orientation arrangements make difference in strain energy densities and total strain energies, (ii) an agreement {a disagreement} between the calculated and the corresponding measured forces values for samples C, D {A, B}. The disagreement might be caused by a greater impact of Ga ion implantation during FIB changing mechanical properties of samples A and B.

## Figures and Tables

**Table 1:** The crystallographic orientations in samples A, B, C, and D, and maximum tensile stresses due to bending.

Cantilever Sample	A	B	C	D
Orientation (in respect to the cantilever top and the cantilever axis)	{0001} <10-10>	{0001} <11-20>	{10-10} <0001>	{10-10} <1-210>
Visualization (cantilever top view, the cantilever axis in the horizontal direction)	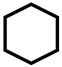	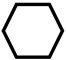	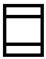	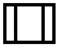
Maximum tensile stress *s*_max_ at maximum bending (MPa)	993 ± 194	1119 ± 178	690 ± 19	663 ± 64

**Table 2:** Geometric parameters of representative samples A, B, C, and D (parameters *L*, *w*, *b*, and *h* are defined in Figure 1b).

Cantilever Sample	*L* (μm)	*w* (μm)	*b* (μm)	*h* (μm)
A	19.3	3.0	2.5	4.2
B	19.2	3.0	2.0	3.7
C	19.9	3.4	2.9	4.8
D	19.3	3.2	2.8	4.7

**Table 3:** The largest Schmidt factors *m*_1_ calculated according to a past paper [26] on the condition of neglecting all the stress components except tension/compression and the corresponding slip/twinning systems for either zone of each cantilever sample.

Cantilever Sample	Tension Zone:		Compression Zone:	Deformation Mode
A	*m*_1_ = 0.433	<a> prismatic	*m*_1_ = −0.5 *	extension twin
B	*m*_1_ = 0.447	<c + a> pyramidal	*m*_1_ = 0.447	<c + a> pyramidal
C	*m*_1_ = 0.5	extension twin	*m*_1_ = 0.447	<c + a> pyramidal
D	*m*_1_ = 0.447	<c + a> pyramidal	*m*_1_ = 0.447	<c + a> pyramidal

*: In the case of compression and twinning, the lowest Schmidt factor matters.

**Table 4:** The calculated elastic strain energy density in the fixed bottom corner, calculated total elastic strain energy, and the calculated {measured} forces belonging microcantilevers A, B, C, and D, assuming deflection equals 0.4 μm.

Microcantilever	A	B	C	D
Calculated elastic strain energy density at the fixed bottom corner [kJ/m^3^]	9860	9850	11,700	12,150
Calculated total elastic strain energy [kJ]	15,000	15,000	16,600	14,900
Calculated {measured} forces corresponding to deflection 0.4 μm [μN]	62{72}	42{57}	104{102}	90{94}
